# Association of frailty status with acute kidney injury and mortality after transcatheter aortic valve replacement: A systematic review and meta-analysis

**DOI:** 10.1371/journal.pone.0177157

**Published:** 2017-05-18

**Authors:** Charat Thongprayoon, Wisit Cheungpasitporn, Natanong Thamcharoen, Patompong Ungprasert, Wonngarm Kittanamongkolchai, Michael A. Mao, Ankit Sakhuja, Kevin L. Greason, Kianoush Kashani

**Affiliations:** 1 Division of Nephrology and Hypertension, Department of Internal Medicine, Mayo Clinic, Rochester, MN, United States of America; 2 Department of Medicine, Bassett Medical Center, Cooperstown, NY, United States of America; 3 Division of Rheumatology, Department of Internal Medicine, Mayo Clinic, Rochester, MN, United States of America; 4 Division of Pulmonary and Critical Care Medicine, Department of Internal Medicine, Mayo Clinic, Rochester, MN, United States of America; 5 Division of Cardiovascular Surgery, Department of Surgery, Mayo Clinic, Rochester, MN, United States of America; University of Florida, UNITED STATES

## Abstract

**Objective:**

Frailty is a common condition in patients with severe aortic stenosis (AS) undergoing transcatheter aortic valve replacement (TAVR). The aim of this systematic review was to assess the impact of frailty status on acute kidney injury (AKI) and mortality after TAVR.

**Methods:**

A systematic literature search was conducted using MEDLINE, EMBASE, and Cochrane databases from the inception through November 2016. The protocol for this study is registered with PROSPERO (International Prospective Register of Systematic Reviews; no. CRD42016052350). Studies that reported odds ratios, relative risks or hazard ratios comparing the risk of AKI after TAVR in frail vs. non-frail patients were included. Mortality risk was evaluated among the studies that reported AKI-related outcomes. Pooled risk ratios (RR) and 95% confidence interval (CI) were calculated using a random-effect, generic inverse variance method.

**Results:**

Eight cohort studies with a total of 10,498 patients were identified and included in the meta-analysis. The pooled RR of AKI after TAVR among the frail patients was 1.19 (95% CI 0.97–1.46, I^2^ = 0), compared with non-frail patients. When the meta-analysis was restricted only to studies with standardized AKI diagnosis according to Valve Academic Research Consortium (VARC)-2 criteria, the pooled RRs of AKI in frail patients was 1.16 (95% CI 0.91–1.47, I^2^ = 0). Within the selected studies, frailty status was significantly associated with increased mortality (RR 2.01; 95% CI 1.44–2.80, I^2^ = 58).

**Conclusion:**

The findings from our study suggest no significant association between frailty status and AKI after TAVR. However, frailty status is associated with mortality after TAVR and may aid appropriate patient selection for TAVR.

## Introduction

Transcatheter aortic valve replacement (TAVR), also known as transcatheter aortic valve implantation (TAVI), has been increasingly utilized for patients with severe aortic stenosis (AS) who are at substantial or prohibitive risk for surgical aortic valve replacement (SAVR) [1–5]. To date, more than 200,000 TAVR procedures have been performed [6, 7]. Although TAVR is considered a less invasive treatment compared with SAVR, the one and two year mortality following TAVR is still considerable at 24% and 34%, respectively [8, 9]. Acute kidney injury (AKI) after TAVR is common with a varied reported incidence ranging from 15% to 57% [2, 6, 10, 11]. Studies have demonstrated significant associations between AKI and decreased survival in patients undergoing TAVR [7, 10, 12].

Frailty is a state of late-life deterioration and vulnerability, characterized by physical weakness, wasting (involving both loss of muscle mass and weight), loss of endurance, decreased balance and mobility, slowed performance, increased sedentary behaviour, and decreased cognitive function [13, 14]. Studies have demonstrated that frailty is associated with adverse health outcomes including postoperative complications, increased hospital length of stay, dependency, falls, discharges to skilled nursing or assisted living facilities, and increased mortality in both general and cardiac surgery populations [15–18].

Among hospitalized patients, a recent retrospective study demonstrated a potential association between frailty and AKI [19]. There is a paucity of data regarding the impact of frailty on AKI incidence, for patients who undergo TAVR. Previous studies of patients undergoing TAVR have not shown such an association [20–27]. Nevertheless, it is possible that previous studies have been underpowered due to sample size. Also, the impact of frailty on AKI after TAVR is unclear due also to the diversity of definitions used for frailty [20–27]. Thus, we conducted this systematic review to assess the impact of frailty on AKI after TAVR comprehensively.

## Materials and methods

### Search strategy

The protocol of this study is registered with PROSPERO (International Prospective Register of Systematic Reviews; no. CRD42016052350). We also followed the Preferred Reporting Items for Systematic Reviews and Meta-Analyses (PRISMA) guidelines (**S1 File**) [28]. Two investigators (CT and WC) systematically searched and reviewed the published literature and conference abstracts indexed in MEDLINE, EMBASE, the Cochrane Central Register of Controlled Trials, and the Cochrane Database of Systematic Reviews from database inception through November 2016 without language restrictions, using the search strategy described in **Text A in S2 File**. Other pertinent references were obtained via manual review of these retrieved references.

### Inclusion criteria

The studies fulfilled the following inclusion criteria: 1) randomized controlled trials (RCTs) or observational studies (cohort, cross-sectional, or case-control studies) published as original articles or conference abstracts that evaluated the risk of AKI after TAVR in frail patients; 2) available data with odds ratio, relative risk, or hazard ratio with 95% confidence intervals (CIs); and 3) a reference group composed of non-frail patients. The primary outcome was AKI after TAVR. Mortality risk was also evaluated among the studies that reported AKI-outcome. Study eligibility was defined by the two investigators noted previously. Differing decisions were solved by mutual consensus. The quality of each study was quantified via the validated Newcastle-Ottawa quality assessment scale for cohort and case-control studies [29] and modified Newcastle-Ottawa scale [30] for cross-sectional studies.

### Data extraction

Two investigators (CT and WC) performed data extraction and analysis of study quality. A standardized data collection template was used to extract the following information: last name of the first author, article title, study design, year of study, country of origin, year of publication, sample size, AKI definition, and definition of frailty.

### Statistical analysis

The data analysis was completed using Review Manager software (Version 5.3, Copenhagen, Denmark) from the Cochrane Collaboration. Point estimates and standard errors were derived from each included study and were combined by the generic inverse variance method of DerSimonian and Laird [31]. Given the likelihood of increased inter-observation variance, a random-effect model was applied. Statistical heterogeneity was evaluated utilizing Cochran’s Q test. These results complemented the I^2^ statistic, which quantifies the proportion of the total variation across studies due to heterogeneity rather than chance. The I^2^ values of <25%, 26%-50%, 51%-75%, and >75% were deemed to represent insignificant, low, moderate, and high heterogeneity, respectively [32]. The presence of publication bias was screened via funnel plots of the logarithm of odds ratios vs. standard errors [33].

## Results

**Fig 1:**
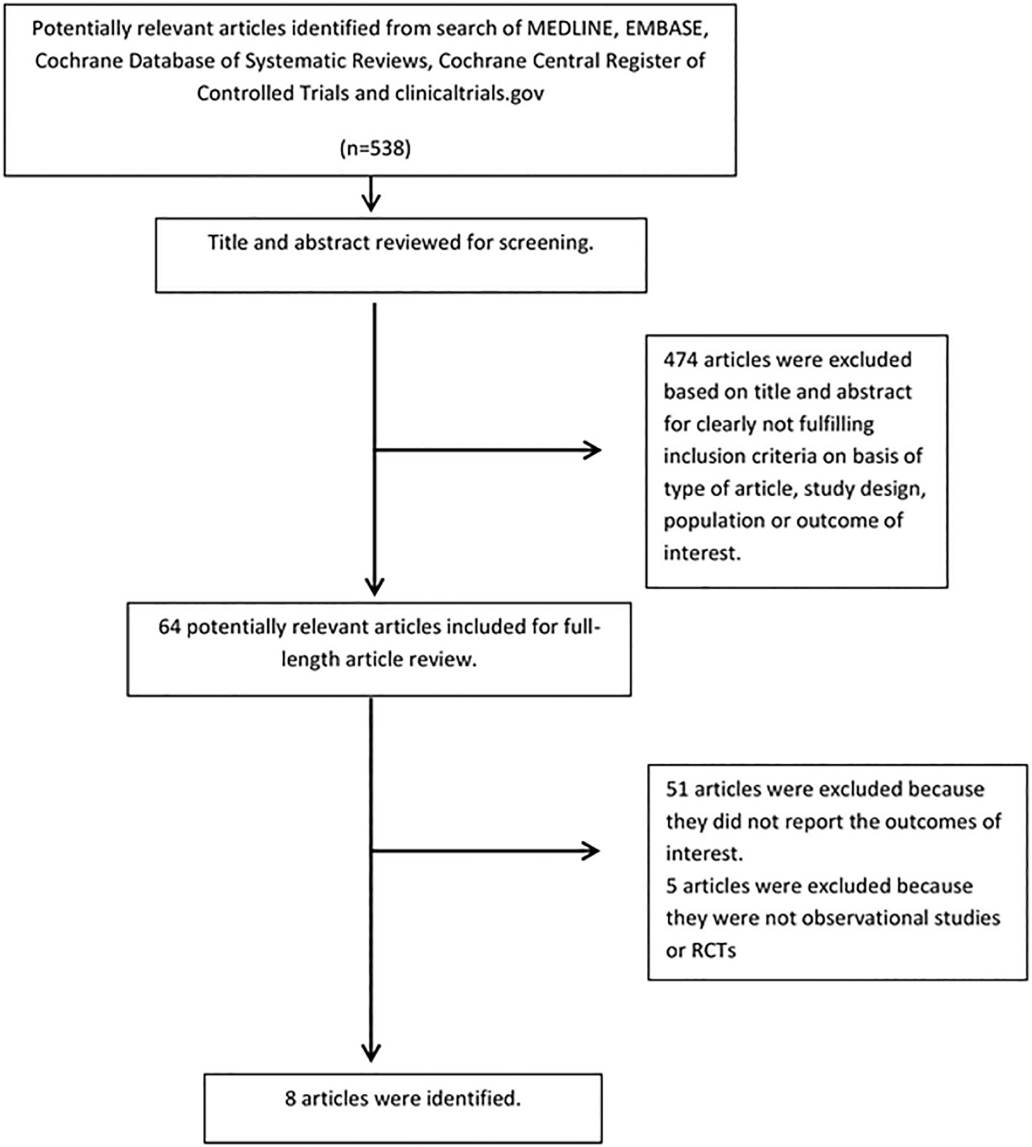
Outline of our search methodology.

Our search strategy yielded 538 articles. Of these, 474 were excluded based on relevance and eligibility criteria following the review of the title and abstract. The remaining 64 articles underwent full-length review, and subsequently, 56 were excluded for failing to meet all eligibility requirements. Of these, 51 articles did not report the outcome of interest, and five articles were not observational studies or RCTs. Eight cohort studies [20–27] with a total of 10,498 patients were included in the meta-analysis to assess the risk of AKI following TAVR in frail vs. non-frail patients (**Table 1**). **Fig 1**outlines our search methodology and selection process.

**Table 1:** Main characteristics of the studies included in this meta-analysis[20–27].

Study	Year	Total number	Frailty definition	AKI definition	Risk ratio for AKI	Risk ratio for mortality	Quality assessment
Green et al[20]	2012	159	Frailty score, based on gait speed, grip strength, serum albumin and activities of daily living status, > 5	Acute kidney injury stage 2 or 3 according to VARC-2 definition	1.10 (0.21–5.60)	3.51 (1.43–8.62)	S 4, C 0, O 3
Stortecky et al[21]	2012	256	BMI < 20 kg/m2	Acute kidney injury stage 3 according to RIFLE definition	1.35 (0.13–13.56)	2.43 (0.92–6.39)	S 4, C 0, O 3
Puls et al[22]	2014	300	Katz index < 6	Acute kidney injury according to modified RIFLE definition	AKI; 1.23 (0.76–2.00), AKI stage 3; 2.23 (1.12–4.47)	2.67 (1.7–4.3)	S 4, C 0, O 3
Green et al[23]	2015	244	Frailty score, based on gait speed, grip strength, serum albumin and activities of daily living status, ≥ 6	Renal failure requiring dialysis	1.62 (0.58–4.49)	2.50 (1.40–4.35)	S 4, C 0, O 3
Yamamoto et al[24]	2015	777	BMI < 20 kg/m2	Acute kidney injury according to VARC-2 definition	0.84 (0.26–2.70)	0.68 (0.29–1.61)	S 4, C 2, O 3
Alfredson et al[25]	2016	8,039	5-m gait speed < 10 s	Acute kidney injury according to VARC-2 definition	1.18 (0.91–1.53)	1.35 (1.01–1.80)	S 4, C 0, O 3
Koifman et al[26]	2016	491	BMI < 20 kg/m2	Acute kidney injury stage 2 or 3 according to VARC-2 definition	1.04 (0.30–3.94)	2.45 (1.26–4.75)	S 4, C 0, O 3
Saji et al[27]	2016	232	The lowest tertile of normalized psoas muscle mass (cross-sectional areas of the psoas muscle at the L4 vertebra level measured by CT and normalized to body surface area)	Acute kidney injury stage 2 or 3 according to AKIN definition	1.20 (0.52–2.80)	2.30 (1.09–4.86)	S 4, C 0, O 3

Abbreviation: AKI, acute kidney injury; AKIN, Acute Kidney Injury Network; BMI, body mass index; S, selection; C, comparability; O, outcome; VARC, Valve Academic Research Consortium.

### AKI definition

All included studies identified AKI occurrence, based on the change in serum creatinine (SCr) or glomerular filtration rate (GFR) after TAVR. Seven [20–22, 24–27] of the eight studies used standard AKI definitions as shown in **Table 1.** Four [20, 24–26] of the eight studies used standardized AKI diagnosis according to Valve Academic Research Consortium (VARC)-2 (definition consisting of the Acute Kidney Injury Network (AKIN) or the Kidney Disease Improving Global Outcomes (KDIGO) criteria with the timing for AKI diagnosis up to 7 days following a TAVR procedure) [34].

### Frailty and AKI risk after TAVR

**Fig 2:**
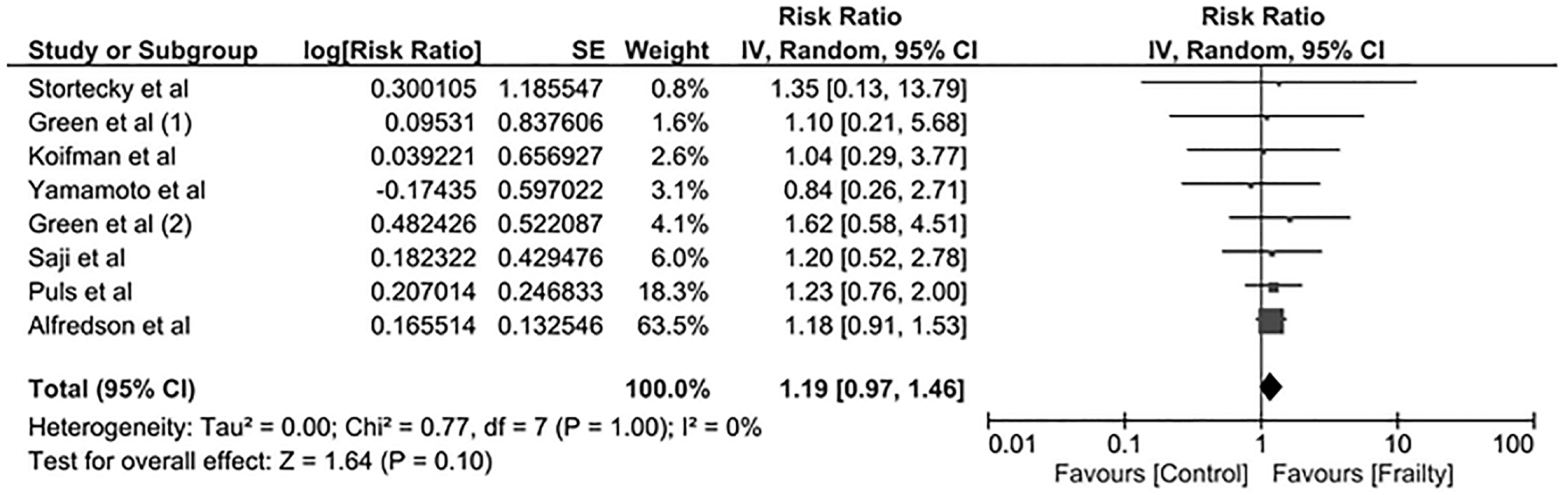
Forest plot of included studies comparing the risk of AKI after TAVR in frail vs. non-frail patients. Square data markers represent risk ratios (RRs), and horizontal lines represent the 95% confidence intervals (CIs) with marker size reflecting the statistical weight of the study using random-effects model. A diamond data marker represents the overall RR and 95% CI for the outcome of interest.

The pooled RR of AKI after TAVR in frail patients was 1.19 (95% CI 0.97–1.46), compared with non-frail patients, as shown in **Fig 2**. The statistical heterogeneity was insignificant with an I^2^ of 0%. When the meta-analysis was restricted only to studies with standardized AKI diagnosis according to VARC-2 criteria, the pooled RRs of AKI in frail patients was 1.16 (95% CI 0.91–1.47, I^2^ = 0).

Of the eight studies, frailty status was identified by frailty score or gait speed in four studies [20, 22, 23, 25] and body mass index (BMI) or psoas muscle mass in the other four studies [21, 24, 26, 27] (**Table 1**). The pooled RR of AKI after TAVR in frail patients based on frailty score, or gait speed was 1.21 (95% CI 0.97–1.51, I^2^ = 0). The pooled RR of AKI after TAVR in frail patients based on BMI or psoas muscle mass was 1.07 (95% CI 0.60–1.93, I^2^ = 0).

### Frailty and mortality risk after TAVR

**Fig 3:**
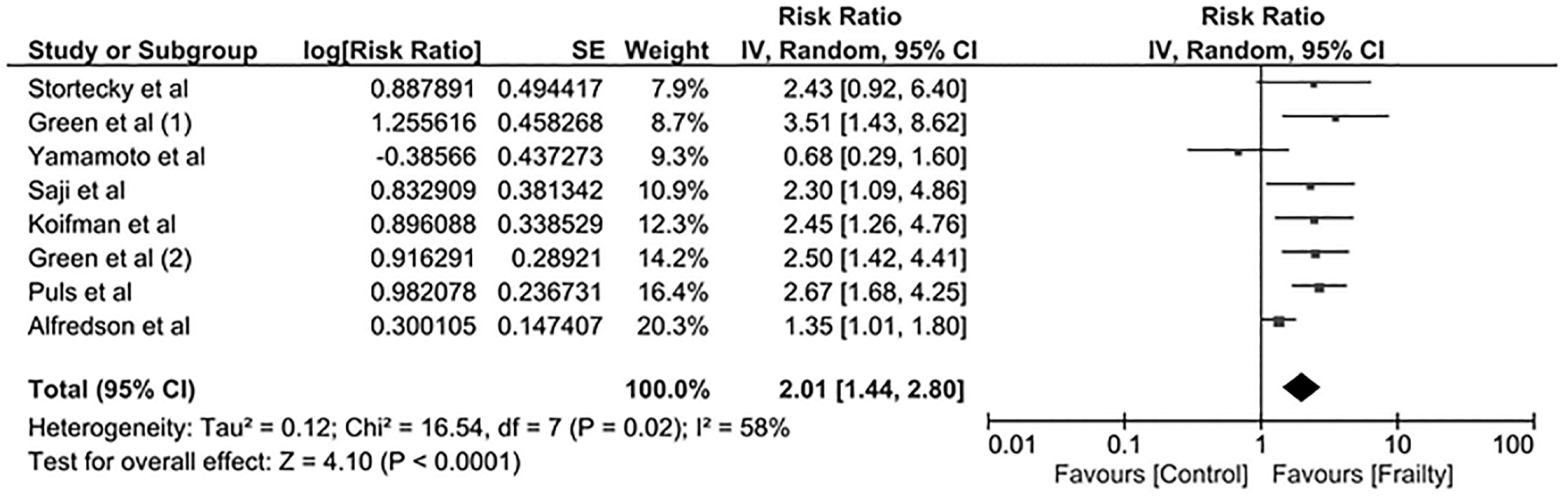
Forest plot of included studies comparing the risk of mortality after TAVR in frail vs. non-frail patients. Square data markers represent risk ratios (RRs); horizontal lines represent the 95% CIs with marker size reflecting the statistical weight of the study using random-effects model. A diamond data marker represents the overall RR and 95% CI for the outcome of interest.

All included studies [20–27] evaluated the association between mortality (within 1 year after TAVR) and frailty status. The pooled RR of mortality in frail patients was 2.01 (95% CI 1.44–2.80, I^2^ = 58), as shown in **Fig 3**. The pooled RR of mortality after TAVR in frail patients based on frailty score or gait speed was 2.19 (95% CI 1.38–3.47, I^2^ = 70). The pooled RR of mortality after TAVR in frail patients based on BMI or psoas muscle mass was 1.78 (95% CI 0.99–3.21, I^2^ = 54).

### Evaluation for publication bias

Funnel plots to evaluate publication bias for the risks of AKI and mortality after TAVR in frail patients are summarized in **Fig A and B in S2 File.** The graphs suggested no significant publication bias.

### Study quality

All included cohort studies were of moderate to high quality [20–27], with a median Newcastle-Ottawa quality assessment scale of 7 (range 7–9) as shown in **Table 1**.

## Discussion

In this systematic review, we demonstrated a statistically insignificant and potentially clinically relevant association between frailty and AKI after TAVR. This association remained insignificant after limiting studies to those that only utilized an AKI diagnosis according to VARC-2 criteria. However, within all included studies, frailty was significantly associated with increased mortality within 1 year in patients undergoing TAVR.

Studies have demonstrated abnormalities in frail patients including immune dysfunction, chronic low-grade systemic inflammation, and endocrine dysregulation such as insulin resistance and testosterone deficiency [13, 35]. Since it is well known that renal ischemia-reperfusion injury incites inflammation that subsequently exacerbates further injury, it has been postulated that frailty in cardiac surgery patients (especially with cardiopulmonary bypass) [36] may predispose patients to AKI [19]. However, based on the findings of this meta-analysis despite higher statistical power, there was no observed significant association between frailty and the risk of AKI after TAVR. These results suggests that the magnitude of frailty impact on AKI after TAVR is likely small compared to other known risk factors for AKI after TAVR (baseline renal function, transapical approach, blood transfusion and the need for circulatory support) [37–42].

Despite an insignificant association between frailty and AKI after TAVR, frail patients still carry a higher mortality risk after TAVR [20–27, 43–46]. Compared with non-frail patients, our meta-analysis demonstrated that frail patients had an approximately two-fold increased mortality risk within one year after TAVR. The increased mortality after TAVR in frail patients is likely multifactorial [15–18], but less likely due to AKI based on the findings of our study. Thus, frailty and AKI may independently affect patients' mortality without significant synergistic effects. Despite limited data on cause of death in the included studies, it has been previously shown that frailty is associated with increased cardiovascular diseases and events that result in increased mortality [47].

Although the studies included in our meta-analysis were all of moderate to high quality, there are some limitations that worth mentioning. First, there are multiple methods to measure or characterize AKI and frailty in the literature [20–27]. VARC-2 standardized the timing for the AKI diagnosis, extending from 72 hours to 7 days following a TAVR procedure using standardized criteria, i.e., the AKIN criteria and KDIGO criteria. Although VARC-2 definition is widely adopted as the standardized endpoint definition for TAVR, these three definitions are very similar with minor differences. Thus, the use of different AKI definition should not result in significant heterogeneity and the findings of our study suggest a statistically insignificant association between frailty and AKI after TAVR in different definitions. The definition of frailty is currently not standardized, and greater than 20 instruments for measurements of frailty have been developed [5]. BMI is used as a simple surrogate for wasting, whereas frailty score might provide more comprehensive functional assessment of the frailty condition [5]. Although there was no significant heterogeneity in our meta-analysis, a standardized definition of frailty and universal frailty assessment tools are needed in order to better understanding the impact of frailty on mortality. Second, there was limited data regarding the cause of death after TAVR. Thus, future studies are warranted to identify the underlying mechanisms on frailty-related mortality after TAVR. In addition, future studies are needed to evaluate if interventions to prevent and improve frailty can reduce mortality after TAVR. Lastly, this is a meta-analysis of observational studies subject to the inherent limitations for which a causal relationship cannot be inferred.

Our meta-analysis indicates no association between frailty and the risk of AKI after TAVR. However, frailty assessment should be part of comprehensive evaluation before TAVR and may aid patient selection process since it is significantly associated with higher mortality in patients undergoing TAVR.

## Supporting information

S1 File PRISMA checklist. (DOC)

S2 File **Text A in S2 File**: Search Strategy
**Fig A in S2 File:** Funnel plot of included studies in the meta-analysis for the risk of AKI after TAVR in frail patients. RR = risk ratio, SE = standard error.
**Fig B in S2 File:** Funnel plot of included studies with adjusted analysis in the meta-analysis for the risk of mortality after TAVR in frail patients. RR = risk ratio, SE = standard error.
(DOC)
