# Supplementary material for: Association of frailty status with acute kidney injury and mortality after transcatheter aortic valve replacement: A systematic review and meta-analysis
Source: PLoS One. 2017 May 18;12(5):e0177157. doi: 10.1371/journal.pone.0177157 (PMC5436661; doi:10.1371/journal.pone.0177157)
Supplement: S2 File — Text A in S2 File: Search Strategy Fig A in S2 File: Funnel plot of included studies in the meta-analysis for the risk of AKI after TAVR in frail patients. RR = risk ratio, SE = standard error. Fig B in S2 File: Funnel plot of included studies with adjusted analysis in the meta-analysis for the risk of mortality after TAVR in frail patients. RR = risk ratio, SE = standard error. (DOC) [file pone.0177157.s002.doc]

**Text A:** Search Strategy

**Database: Ovid, MEDLINE, Cochrane Database of Systematic Reviews, Cochrane Central Register of Controlled Trials and Clinicaltrials.gov**

1. Transcatheter Aortic Valve.mp
2. exp TAVI/
3. TAVI.mp
4. TAVR.mp
5. Percutaneous Valve.mp
6. Transcutaneous Aortic Valve.mp
7. Transcatheter Aortic Valve.mp
8. 1 or 2 or 3 or 4 or 5 or 6 or 7
9. frailty.mp
10. exp frail/
11. frail.mp
12. 9 or 10 or 11
13. 8 and 12

**Fig A:** Funnel plot of included studies in the meta-analysis for the risk of AKI after TAVR in frail patients. RR = risk ratio, SE = standard error.

**
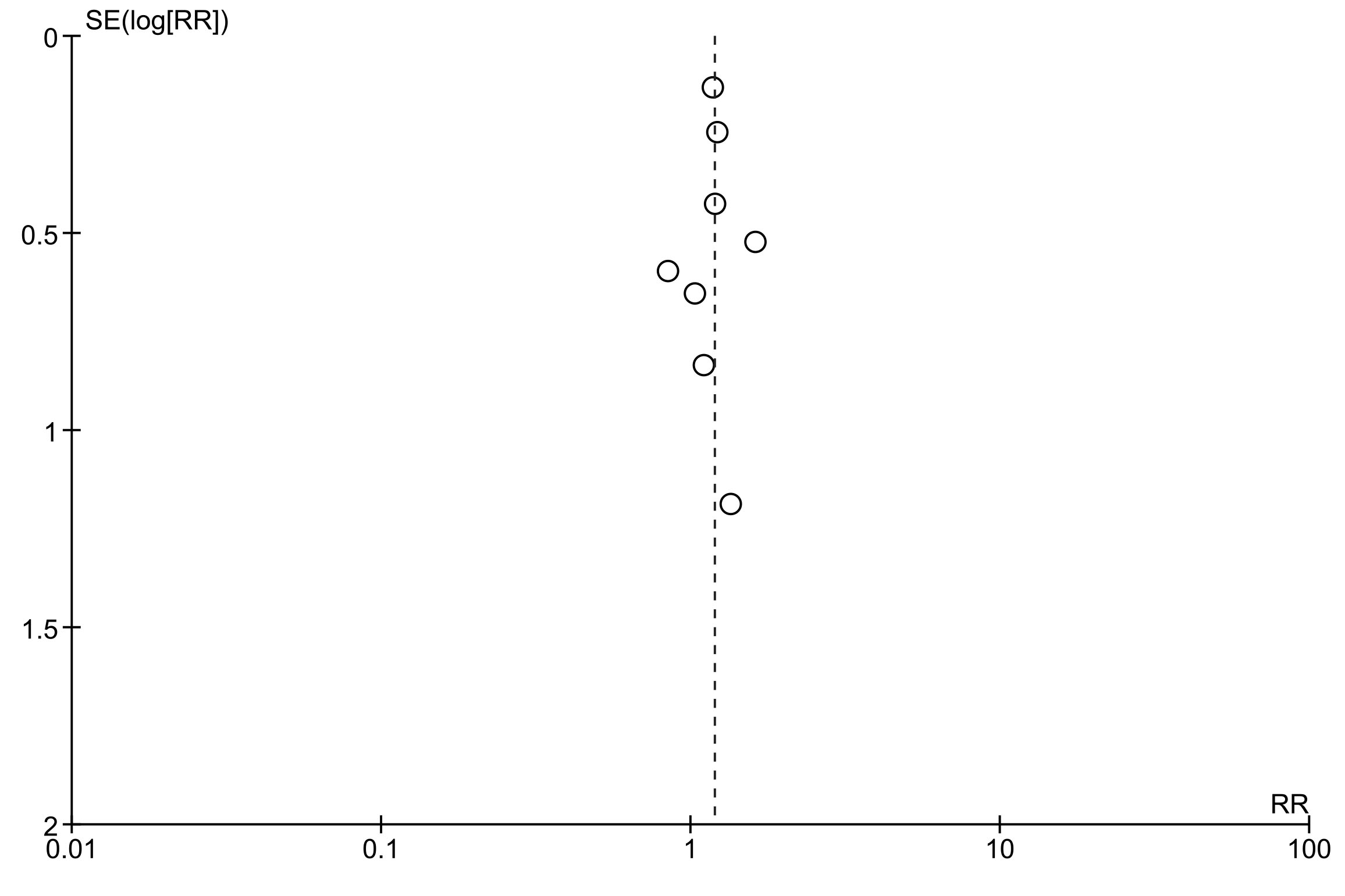
**

**Fig B:** Funnel plot of included studies with adjusted analysis in the meta-analysis for the risk of mortality after TAVR in frail patients. RR = risk ratio, SE = standard error.

**
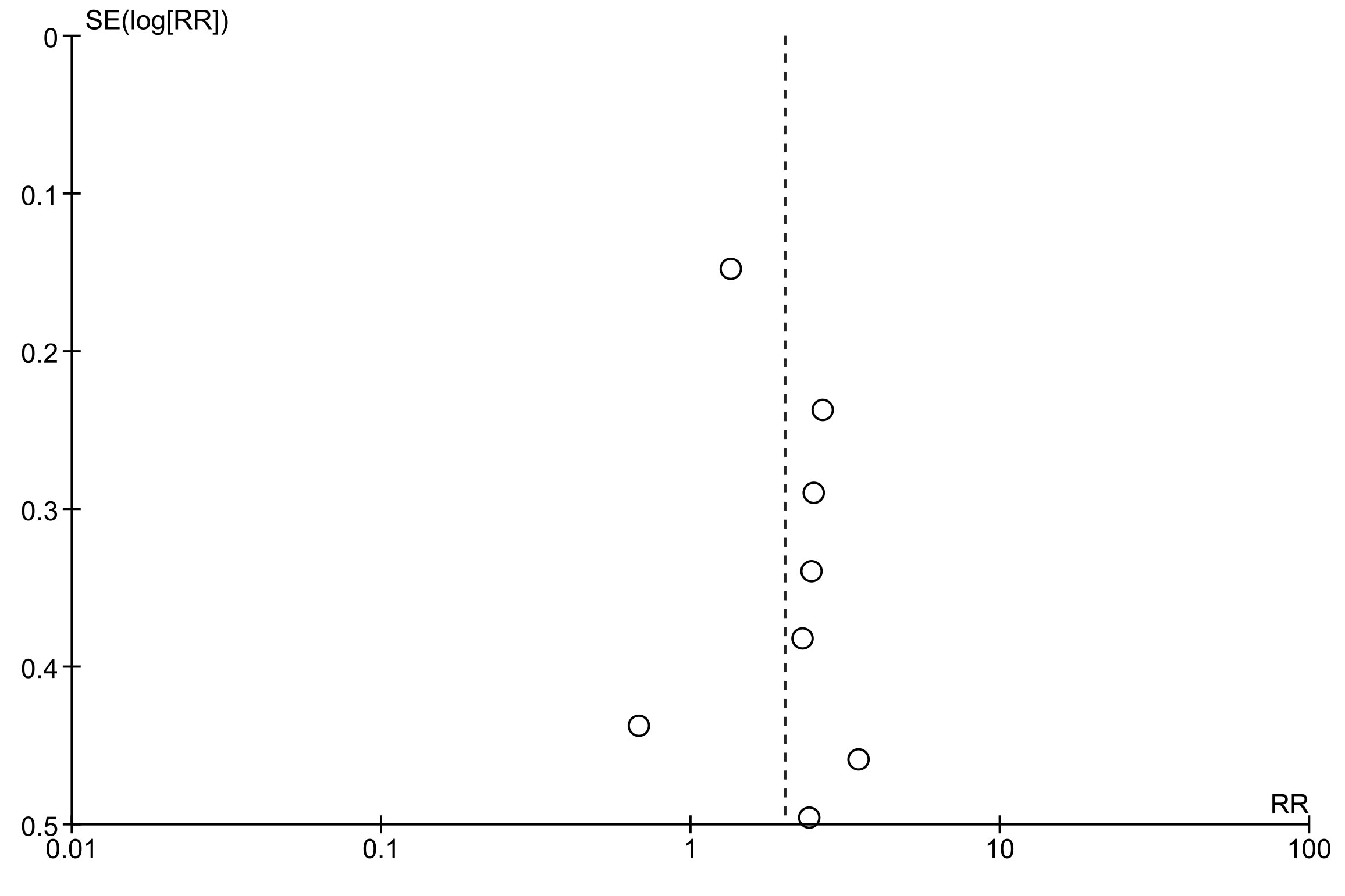
**
